# Age-adjusted cut-off values of lipid accumulation product (LAP) for predicting hypertension

**DOI:** 10.1038/s41598-021-90648-y

**Published:** 2021-05-27

**Authors:** Anastasiya M. Kaneva, Evgeny R. Bojko

**Affiliations:** Institute of Physiology of Кomi Science Centre of the Ural Branch of the Russian Academy of Sciences, FRC Komi SC UB RAS, 50 Pervomayskaya str, 167982 Syktyvkar, Russia

**Keywords:** Physiology, Biomarkers, Diseases, Cardiovascular diseases

## Abstract

Among the many factors considered relevant to hypertension, obesity and metabolic disturbances play an important role in the development of this pathology. Therefore, lipid accumulation product (LAP), an index of visceral adiposity, is a simple and effective indicator of hypertension risk. To date, the reference and cut-off values for LAP have not been defined. The aim of the study was to determine the age-adjusted optimal cut-off values of LAP for the prediction of hypertension risk. This cross-sectional case–control study comprised 1960 subjects ranging from 20 to 64 years of age. The participants underwent anthropometric tests, blood pressure measurements, questionnaire surveys and laboratory examinations. The cut-off values of LAP were determined using receiver operating characteristic (ROC) curve analysis. According to our study results, LAP values in healthy subjects increased with age, whereas there was no effect of age on LAP values in patients with hypertension. These two findings determine the presence of age-adjusted cut-off values of LAP for diagnosing hypertension. Increasing age is associated with an increase in the cut-off values of LAP to detect hypertension. In conclusion, hypertension risk should be estimated using the age-adjusted cut-off values of LAP; otherwise, the risk of hypertension might be overestimated or underestimated.

## Introduction

Lipid accumulation product (LAP) is a current marker of visceral obesity and is based on a combination of two variables, waist circumference (WC) and fasting concentration of triglycerides (TG). The simultaneous use of biochemical and anthropometric measurements to calculate LAP allows to describe both anatomical and biochemical changes related to lipid overaccumulation in humans^1^. LAP is widely used as a marker of metabolic disorders and a predictor of cardiovascular diseases^2^.

Hypertension is the most common cardiovascular disease. The main risk factors for hypertension are age, sex, family history, genetic composition, unhealthy diets, excessive salt intake, the consumption of tobacco and alcohol, physical inactivity and obesity^3^. At present, hypertension is assumed to be associated with metabolic disturbances and may be considered a metabolic disorder^4^. Thus, LAP could be a good marker to predict hypertension because it is classified as a mixed index calculated from WC and fasting TG levels, which reflect accumulated and circulating fat, respectively.

The ability of LAP to predict or detect prehypertension and hypertension has been demonstrated in many previous studies^5–8^. However, the optimal LAP cut-off point for identifying hypertension has not yet been determined. The cut-off values of LAP for hypertension obtained in some studies are very different. Reported LAP cut-off values ranged from 25.16 to 31.59 cm mmol/l for women and from 20.10 to 63.89 cm mmol /l for men^9–12^. These discrepancies may be due to differences in sample populations with regard to age range, race/ethnicity, criteria for screening hypertension, and health status of the control group. Age seems to have the greatest impact on the optimal LAP cut-off points for predicting hypertension because of the effect of age on LAP values^13–17^.

The aim of the study was to determine the age-adjusted optimal cut-off values of LAP for the prediction of hypertension risk.

## Materials and method

### Study population

The study included 1960 subjects (1052 women and 908 men aged 20 to 64 years). Of these, 1505 participants were apparently healthy, and 435 patients had hypertension but no other concomitant diseases (e.g., cardiovascular diseases, stroke and diabetes mellitus). The diagnosis of hypertension in patients was based on clinical history. Healthy participants without known hypertension but whose systolic blood pressure or diastolic blood pressure was higher than 140 mmHg or 90 mmHg, respectively, at the time of the study were not included because they were considered to be individuals with possible latent hypertension. Subjects were divided into three age subgroups, spanning 15 years each.

Information regarding personal characteristics, lifestyle factors, health status and medication history was obtained by a staff-administered standardized questionnaire. Each subject gave written informed consent for participating in this study, which was conducted under the guiding principles of the Declaration of Helsinki and was approved by the local ethics committee of Institute of Physiology of Кomi Science Centre of the Ural Branch of the Russian Academy of Sciences.

### Measurements

Anthropometric measurements were made by well-trained examiners using standardized instruments. Body weight was measured on a digital scale with participants wearing light clothing and no shoes. Height was determined without shoes using a standard stadiometer. Body mass index was calculated as weight (kg) divided by height squared (m2). WC (cm) was measured using nonelastic tape at the midway point between the last rib and the iliac crest after normal expiration. Blood pressure was measured on the right arm using an automated device (DINAMAP-R (Criticon, Tampa, Florida)) after the participant rested for 10 min in a seated position. Blood pressure was measured 3 times with a 3-min interval between each measurement, and the average of the three measurements was recorded.

### Biochemical analyses

Blood samples were collected from venous vessels after 12-h fasting. Total cholesterol (TC), TG and high-density lipoprotein cholesterol (HDL-C) levels were measured using an enzymatic method with commercially available kits. HDL-C concentration was determined by assaying the cholesterol in the supernatant obtained after the precipitation of apoB-containing lipoproteins with phosphotungstate/magnesium chloride.

The non-HDL-C was calculated as the difference between TC and HDL-C. Atherogenic index of plasma (AIP) was calculated as the logarithm of the ratio of the molar concentration (mmol/L) of TG to HDL-C (i.e., log [TG/HDL-C])^18^. LAP values were determined using fasting TG levels (mmol/L) and WC (cm) as follows: LAP = (WC-58) × TG for women; LAP = (WC-65) × TG for men^1^.

### Statistical analysis

Data were analysed using Statistica 8.0 (Statsoft, Tulsa, USA) and MedCalc 19.5.1 (MedCalc Software Ltd, Belgium). Continuous variables are presented as the median and 25th and 75th percentiles. Categorical variables are presented as absolute values (n) and percentages (%). Differences between groups were compared using Mann–Whitney tests, Kruscal-Whallis test (with multiple comparisons by Dunn test) and chi-square tests. Receiver operating characteristic (ROC) curve analysis was used to determine the age-adjusted cut-off values of LAP for defining hypertension risk. A value of *p* < 0.05 was accepted as statistically significant.

## Results

A total of 1960 subjects (1052 women and 908 men) participated in the present study, including 455 patients (282 women and 173 men) confirmed as having hypertension. The baseline characteristics of the participants, grouped by hypertension status, are shown in Table 1.

**Table 1:** Baseline characteristics of participants with and without hypertension.

Characteristics	Women, n = 1052			Men, n = 908		
	Normotension n = 770	Hypertension n = 282	*p* Value	Normotension n = 735	Hypertension n = 173	*p* Value
Age, years	37.0 (28.0; 45.0)	49.5 (43.0; 55.0)	** < 0.001**	38.0 (30.0; 47.0)	50.0 (43.0; 55.0)	** < 0.001**
Weight, kg	64.0 (57.0; 71.0)	73.0 (64.0; 85.0)	** < 0.001**	74.0 (67.0; 81.0)	82.0 (74.0; 93.0)	** < 0.001**
Height, cm	164.0 (159.0; 167.0)	161.0 (157.0; 165.0)	** < 0.001**	175.0 (170.0; 180.0)	173.0 (168.0; 178.0)	**0.009**
Body mass index, kg/m2	23.6 (21.5; 26.7)	28.3 (24.7; 32.3)	** < 0.001**	24.4 (22.1; 26.5)	27.5 (25.0; 30.3)	** < 0.001**
WC, cm	73.0 (68.0; 82.0)	87.0 (79.0; 96.0)	** < 0.001**	81.0 (76.0; 88.0)	90.0 (84.0; 98.0)	** < 0.001**
LAP, cm mmol /l	13.8 (7.7; 26.6)	41.2 (23.0; 69.1)	** < 0.001**	18.9 (9.8; 32.1)	34.7 (20.3; 51.5)	** < 0.001**
SBP, mm Hg	122.0 (114.0; 130.0)	148.0 (135.0; 165.0)	** < 0.001**	129.0 (123.0; 136.0)	154.0 (142.0; 167.0)	** < 0.001**
DBP, mm Hg	69.0 (62.0; 76.0)	85.0 (76.0; 95.0)	** < 0.001**	72.0 (65.0; 79.0)	90.0 (81.0; 101.0)	** < 0.001**
TC, mmol/l	5.05 (4.34; 5.85)	5.87 (4.94; 6.67)	** < 0.001**	5.23 (4.52; 6.03)	5.67 (4.96; 6.48)	** < 0.001**
TG, mmol/l	0.94 (0.71; 1.30)	1.44 (0.96; 2.06)	** < 0.001**	1.17 (0.86; 1.64)	1.34 (1.01; 1.93)	** < 0.001**
HDL-C, mmol/l	1.26 (1.12; 1.37)	1.30 (1.16; 1.44)	** < 0.001**	1.23 (1.10; 1.34)	1.21 (1.10; 1.34)	0.419
TC/HDL-C	4.02 (3.45; 4.67)	4.43 (3.82; 5.19)	** < 0.001**	4.25 (3.71; 5.05)	4.77 (4.01; 5.36)	** < 0.001**
AIP	-0.11 (-0.24; 0.03)	0.03 (-0.13; 0.22)	** < 0.001**	-0.02 (-0.15; 0.13)	0.05 (-0.08; 0.21)	** < 0.001**
non-HDL-C, mmol/l	3.76 (3.09; 4.53)	4.52 (3.67; 5.36)	** < 0.001**	3.99 (3.34; 4.78)	4.43 (3.72; 5.20)	** < 0.001**

Bold values indicate *p* < 0.05

Data are presented as the median and interquartile range (25th and 75th percentile). Mann–Whitney test was used to estimate differences between the groups.

WC, waist circumference; LAP, lipid accumulation product; SBP, systolic blood pressure; DBP, diastolic blood pressure; TC, total cholesterol; TG, triglycerides; HDL-C, high-density lipoprotein cholesterol; AIP, atherogenic index of plasma.

In both women and men, there were significant differences for almost all baseline variables according to hypertension status. Hypertensive patients were older than normotensive subjects. The mean values of anthropometric indices and blood pressure were significantly higher in women and men with hypertension than in those without hypertension (*p* < 0.001). Lipid levels and indices were also higher among patients with hypertension (*p* < 0.001), except for HDL-C in men.

Table 2 provides LAP values for subjects with and without hypertension separately according to age subgroups (20–34, 35–49, and 50–64 years). In normotensive subjects, the mean values of LAP in the whole group were lower in women than in men (*p* < 0.001). LAP values rose with an increase in age for both sexes, but the rate of this rise was different for women and men. This feature resulted in LAP values being significantly lower in women than in men in younger age subgroups (20–34 and 35–49 years) and did not differ significantly in the oldest subgroup (50–64 years).

**Table 2:** LAP values according to age subgroups in women and men with and without hypertension.

	Normotensive women			Normotensive men			*p* Value^1^
	n	Median (25%; 75%)	Min–Max	n	Median (25%; 75%)	Min–Max	
20–64	770	13.8 (7.7; 26.6)	0.4–133.2	735	18.9 (9.8; 32.1)	0.5–156.5	** < 0.001**
20–34 (1)	336	8.7 (4.6; 15.1)	0.4–90.2	285	13.3 (7.2; 24.0)	0.5–130.4	** < 0.001**
35–49 (2)	325	18.5 (11.0; 30.4)***^1–2^	0.4–133.2	315	21.4 (12.3; 33.2)***^1–2^	1.8–156.5	**0.03**
50–64 (3)	109	33.2 (18.6; 47.3)***^1–3;2–3^	4.80–132.7	135	26.2 (17.0; 44.3)*^2–3;^***^1–3^	2.2–123.5	0.106
p-value^2^	** < 0.001**			** < 0.001**			
	Hypertensive women			Hypertensive men			
	n	Median (25%; 75%)	Min–Max	n	Median (25%; 75%)	Min–Max	
20–64	282	41.2 (23.0; 69.1)^###^	1.0–264.1	173	34.7 (20.3; 51.5)^###^	2.1–271.3	**0.010**
20–34 (1)	24	30.9 (12.7; 66.7)^###^	2.1–130.6	19	30.2 (14.8; 50.3)^##^	3.5–99.4	0.807
35–49 (2)	117	43.2 (22.4; 70.6)^###^	1.0–264.1	67	42.5 (23.3; 60.6)^###^	3.5–271.3	0.698
50–64 (3)	141	42.9 (26.9; 65.0)^###^	5.0–243.0	87	31.7 (18.8; 45.0)	2.1–164.9	**0.001**
p-value^2^	0.188			0.089			

Bold values indicate *p* < 0.05

^1^The statistical significance of differences between men and women was estimated using the Mann–Whitney test.

^2^The statistical significance of differences between age groups was estimated using the Kruskal–Wallis test. Differences between age groups are statistically significant at *p < 0.05 and ***p < 0.001 (multiple comparisons by Dunn test). ##*p* < 0.01; ###*p* < 0.001 – The statistical significance of differences between normotensive and hypertensive subjects within each corresponding age group.

In both women and men, LAP values were substantially higher in subjects with hypertension than in those without hypertension. There was no effect of age on LAP values in patients with hypertension.

ROC curve analysis was performed to establish cut-offs of LAP to determine hypertension. The area under the ROC curve (AUROC) value of LAP for defining hypertension in the whole study group of women was 0.785 (95% CI 0.754–0.816), and the cut-off value of LAP was 27.0 cm mmol /l with 70.6% sensitivity and 75.8% specificity (Table 3). The cut-off value of LAP for assessing incident hypertension risk in women younger than 35 years of age was less than that in the whole group of women. The LAP cut-off point in middle-aged women was equal to that in the general group of women. In the older-age subgroup, the cut-off value of LAP was above than that in the whole group of women.

**Table 3:** The areas under the ROC curve (AUROCs), optimal cut-off values, sensitivities and specificities for LAP associated with hypertension in men and women according to age subgroups.

Age (year)	AUROC (95% CI)	Cut-off point	Sensitivity (%)	Specificity (%)	*p* Value
Women					
20–64	0.785 (0.754–0.816)	27.0	70.6	75.8	** < 0.001**
20–34	0.801 (0.701–0.902)	13.8	75.0	73.5	** < 0.001**
35–49	0.735 (0.679–0.791)	27.0	69.2	72.3	** < 0.001**
50–64	0.624 (0.555–0.693)	44.6	46.8	72.5	** < 0.001**
Men					
20–64	0.694 (0.650–0.738)	27.6	63.6	68.3	** < 0.001**
20–34	0.715 (0.571–0.858)	24.0	68.4	74.7	**0.003**
35–49	0.712 (0.641–0.783)	36.4	61.2	78.4	** < 0.001**
50–64	0.551 (0.474–0.629)	26.7	64.4	53.3	0.193

Bold values indicate *p* < 0.05

AUROC, area under the receiver-operating characteristic curve; CI, confidence interval.

The AUROC value of LAP for predicting hypertension in the whole study group of men was relatively smaller than that in women (0.694, 95% CI 0.650–0.738) (Table 3). The LAP cut-off value for predicting hypertension in the whole group of men was 27.6 cm mmol /l, with 63.6% sensitivity and 68.3% specificity. The cut-off value of LAP in the age subgroup below 35 years was slightly lower than that in the general group of men. Men from the middle-age subgroup had a higher cut-off value, while older men had a value that was similar to that in the whole group of men.

As seen in Table 3, a significant effect of age on the accuracy of LAP values for identifying hypertension was found. The AUROC values decreased from 0.801 (20–34 years) to 0.624 (50–64 years) in women and from 0.715 (20–34 years) to 0.551 (50–64 years) in men. For men older than 50 years, the confidence intervals for the AUROC included 0.5; thus, there was no predictive performance of LAP for the prediction of hypertension risk. Overall, the ROC curve analysis revealed that LAP had better diagnostic power for predicting hypertension in women than in men for each age subgroup.

## Discussion

The main finding of this study is that the optimal cut-off values of LAP for diagnosing hypertension vary with age. This finding is because values of LAP in healthy subjects increased with age, whereas there was no effect of age on LAP values in patients with hypertension. Age-related changes in LAP values in healthy subjects were primarily associated with a conventional age increase in WC and TG applied for the calculation of this index. The absence of age-related changes in LAP values in patients with hypertension indicates the early development of metabolic disorders manifested by the accumulation of lipids in the abdominal adipose tissue or an increase in the TG level in circulating blood. Only a limited number of population-based studies have estimated the effect of age on LAP values^13–17^. In addition, the designs of these studies varied greatly. Nevertheless, LAP values, as a rule, tend to increase with age. The effect of age on LAP values in patients with hypertension has not been previously studied.

According to a number of studies, in the general adult population, there is great variability in the cut-off values of LAP to define hypertension. Song et al. noted that the best thresholds of LAP to predict hypertension were 29.14 cm mmol /l in women and 40.60 cm mmol /l in men^9^. Huang et al. showed that the cut-off values of LAP for identifying hypertension were 30.86 cm mmol /l in women and 63.89 cm mmol /l in men^11^. In these two studies, the cut-off points of LAP were higher in men than in women. At the same time, other authors demonstrated that the LAP cut-off values for the identification of hypertension were higher in women than in men (25.16 and 20.10 cm mmol /L in women and men, respectively^10^; 31.59 and 21.48 cm mmol/L in women and in men, respectively^12^). Our research indicated that in the whole study group, the cut-off points of LAP for the prediction of hypertension were 27.0 cm mmol /l in women and 27.6 cm mmol/l in men.

High variability in the cut-off values of LAP for identifying hypertension may be associated with significant differences in the age range of the studied populations. Meanwhile, the effect of age on the cut-off values of LAP has not been previously analysed. Our study was the first to determine the age-adjusted cut-off values of LAP for defining hypertension. The cut-off values of LAP for identifying hypertension increased with increasing age. Lower cut-off values of LAP in younger age subgroups may indicate that lipid over accumulation has a greater impact on hypertension risk in younger subjects than in older subjects. This finding is in accordance with data that showed that the pathophysiology of hypertension in young and middle-aged adults differs from that in elderly subjects and tends to be associated with metabolic disorders^19^. Whereas, the age-associated increase in large-artery stiffness is the main characteristic of arterial hypertension in elderly individuals^20^. Another important mechanisms contributing to the development of arterial hypertension in elderly individuals are mechanical haemodynamic changes, endothelial dysfunction, neurohormonal and autonomic dysregulation, and the ageing kidney^21^.

Overall, in both sexes, the ability of LAP to discriminate the presence of hypertension with age decreased. This fact is not consistent with the data of Nguyen et al., who did not reveal an influence of age on the association of LAP with hypertension^12^. Meanwhile, a dependence of the diagnostic accuracy of LAP on age was found for nonalcoholic fatty liver disease (NAFLD) and diabetes^14,22^. In these studies, the accuracy of LAP as a marker for NAFLD and diabetes was significantly better in the younger age groups than in the older age groups.

Increasing age is associated with an increase in the cut-off values of LAP for identifying hypertension. This effect was more pronounced in women than in men. The use of LAP cut-off values determined without distinction for age may lead to an underestimation of hypertension risk in younger age groups and an overestimation of hypertension risk in older age groups.

An incorrect estimation of hypertension risk may have significant implications on the clinical management of patients. The danger of arterial hypertension lies in the asymptomatic course, which does not bother the patient, and the absence of a clear effect on the quality of life before complications develop. The underestimation of hypertension risk leads to missed therapeutic and preventive opportunities. The early detection and prevention of hypertension, especially among young people, could effectively reduce hypertension progression and the risk of developing cardiovascular disease. The overestimation of hypertension risk results in additional costs, unnecessary treatment and potential adverse medication effects.

### Strengths and limitations

The strengths of this study are as follows. Our study is the first to determine the age-adjusted cut-off values of LAP for the prediction of hypertension risk. We carefully selected the group of patients with hypertension. This group included only clinically diagnosed patients.

This study has some limitations. First, the cross-sectional nature of our study does not allow us to explain the reason for the absence of age-related effects on LAP values in hypertensive patients. Second, this study enrolled a relatively small sample size of hypertensive patients. The third limitation is that this study was conducted only in patients with hypertension without other comorbidities. Therefore, the obtained age-adjusted cut-off values of LAP might not be valid for hypertensive subjects with other diseases. Fourth, we did not use 24 h ambulatory blood pressure monitoring to more accurately detect masked hypertension in health subjects. Finally, the study population was composed of participants aged 20 to 64 years; thus, the results cannot be generalized to subjects who are younger or older than this age range.

## Conclusion

LAP is an index of visceral adiposity that predicts hypertension risk. Age affected the optimal LAP cut-off points to assess hypertension risk. LAP cut-off values for the diagnosis of hypertension were lowest in young subjects and then increased with age. The use of age-adjusted cut-off values of LAP will avoid the underestimation or overestimation of hypertensive risk and may improve the prognostic stratification of patients with hypertension.

## Data Availability

The data that support the findings of this study are available from the corresponding author, [A.M.K.], upon reasonable request.
